# Predicting the availability of power line communication nodes using semi-supervised learning algorithms

**DOI:** 10.1038/s41598-025-01064-5

**Published:** 2025-05-21

**Authors:** Kareem Moussa, Khaled Mostafa Elsayed, M. Saeed Darweesh, Abdelmoniem Elbaz, Ahmed Soltan

**Affiliations:** 1 University of Science and Technology, Zewail City, Giza 12578 Egypt; 2 Wireless Intelligent Networks Center (WINC), Nile University, Giza, 12677 Egypt; 3 Faculty of Computers and Artificial Intelligence, Cairo University, Giza, Egypt; 4 School of Engineering and Applied Sciences, Nile University, Giza, 12677 Egypt; 5 El Sewedy Electrometer Group, Al Jizah, Egypt; 6 Nanoelectronics Integrated Systems Center (NISC), Nile University, Giza, 12677 Egypt

**Keywords:** Power Line Communication (PLC), Machine Learning, Semi Supervised Learning, Self Training Classifier, Light Gradient Boosting Machine (LGBM), Support Vector Machine (SVM), Label Propagation, Label Spreading, Mathematics and computing, Electrical and electronic engineering

## Abstract

Power Line Communication (PLC) facilitates the usage of power cables to transmit data. The issue is that sending data to unavailable nodes is time-consuming. Machine Learning has solved this by predicting a node having optimum readings. The more the machine learning models learn, the more accurate they become, as the model becomes always updated with the node’s continuous availability status, so self-training algorithms have been used. A dataset of 2000 instances of a node of a 500-node implemented PLC network has been collected. These instances consist of CINR(Carrier-to-Interference plus Noise Ratio), SNR(Signal-to-Noise Ratio), and RSSI(Received Signal Strength Indicator) as features for the label, which is a node is UP/Down. The data set has been split into 85% as a training set and 15% as a testing set. 15% of the training data are unlabeled. Self-training classifier has been used to allow Light Gradient Boosting Machine (LGBM) and Support Vector Machine (linear and non-linear kernel) to behave in a self-training manner as well as the training of label propagation and label spreading algorithms. Supervised Learning algorithms (Random Forest and logistic regression) have been trained on the dataset to compare the results. The best model is the Label Spreading, which resulted in accuracy equals 94.67%, f1-score equals 0.947, precision is 0.946, and recall equals 0.947 with training time equals 0.018 sec. and memory consumption equals 0.99 MB.

## Introduction

In Power Line Communication, data is transferred using the power grid^1^. It is applied in several applications, including smart grids, industrial automation, appliance control, and home lighting^2,3^. The data is transmitted in several stages. The sender has the data modulated to a high-frequency signal. Then, a coupling capacitor and combined filters load the signal to the transmission line. The receiver demodulates the signal^4^. PLC is categorized into two categories, which are Narrow-band and Broadband^5^. Narrow-band has a frequency range of 3 to 500 kHz^6^. The frequency of the broadband ranges from 1.8 to 250 MHz^7^.

The power line communication transmission faces difficult conditions like high interference and low working conditions. A PLC node becomes unavailable due to several factors, including the noise caused by appliances that reduce the SNR, network collisions, and power outages. The nonuniform distribution of the power lines and different load equipment types at the branch points cause attenuation characteristics^8–12^.

PLC nodes may not be in their ideal condition to be read properly. The cost of sending data to an unavailable node is high. Machine learning can be used in detecting the availability of a node. Machine learning is allowing the computer to learn through a pattern. Supervised learning algorithms and Unsupervised Learning are two types of machine learning. In supervised learning, the computer is given a set of features and a mapped vector of the labels. The machine can learn by adjusting its weight to fit the pattern in the given data^13,14^. In unsupervised learning, the data is given to the computer unlabeled with no classes, and the computer model detects the patterns between the data and groups them into clusters^15,16^.

Applying artificial intelligence in predicting the availability of power line communications nodes. The authors of^17^have conducted a comparative study between several machine learning algorithms representing the statistical, regression, Vector-based, predictive, and decision algorithms. They have trained adaptive boosting, the Support Vector Machine (SVM) linear kernel and the SVM non-linear kernel, the random forest and decision trees, K-Nearest Neighbors on a dataset consisting of 1000 readings based on their SNR, CINR, and RSSI with labels 0 for down and 1 for up. The results reached were the ADA algorithm reaching 0.86613, 87%, 0.8646, and 0.9 for the f1-score, accuracy, recall, and precision respectively. The authors of^18^applied clustering, which naturally groups similar data into clusters depending on metrics for similarity^19^. They applied the clustering algorithms on the MIMO NB noise database to check the usefulness of automatic clustering of the PLCs’ multi-conductor noise. They created a feature library. Box Plots and Principal Component Analysis (PCA) have been used to evaluate the features to determine which features are worth to be considered. Box plot summarizes the data on a graph based on six metrics, which are the minimum, median, 25 th and 75 th percentiles, outlier, and maximum. It showed that the data of the feature 5, which is the Samples Skewness, and the feature 7 which is the Samples Pearson correlation are visibly separated. Hierarchical clustering, CURE clustering, which refers to clustering using representatives, and self-organizing map (SOM) have been used in clustering. Hierarchical clustering groups each data point in a separate cluster. Then the distance between each of the two clusters is calculated, and the closest two clusters are grouped in a cluster, and this process is iterated till all the points are in one cluster, forming a tree of clusters called a dendrogram^20,21^. In CURE clustering, the data are clustered initially, and then representative points for each cluster that are far from each other are shrunk by being moved 20% towards the centroid, and then the nearby clusters are grouped^22,23^. In SOM, a network of nodes is formed whose weights are being updated, allowing the nodes to be more similar to the represented data till they reach a map that shows the data clustered according to the similarity^24,25^. The data has been clustered, and the clusters have been labeled according to the probability density to be 35% normal, 23% Middleton Class A, 13% Generalized Extreme Value, 27% Alpha Stable, and 2% unknown.

The conditions of a PLC node may differ throughout time due to the surrounding conditions, which suggests the continuously of the learning of the model to better predict the node availability which is occur in the semi-supervised learning. It is giving the computer, some labeled data and some unlabeled data such that it learns from the labeled data and according to this training, it labels the unlabeled data, and the newly labeled data with high confidence are considered part of the labeled subset of the data and retrain the model and then label new data and so on^26–28^. A dataset has been collected in this work. It consists of 2000 instances of node readings in a 500-node network, which were implemented by Microchip technology PL360 PLC transceiver using the PRIME standard. Self-training, semi-supervised learning approach is proposed to predict the availability of a power line communication node by training and comparing between Supervised learning algorithms (Random Forest and Logistic Regression) and self-training classifiers which are light gradient boosting machine, support vector machine (linear and non-linear kernels), label propagation, and label spreading on a PLC node availability collected dataset by considering the readings of the Carrier to Interference-plus-Noise (CINR), Signal to Noise Ratio (SNR), and Received Signal Strength Indicator (RSSI). Predicting the availability of a node saves time and power due to the high cost of sending data to an unavailable node.

The model details, the dataset, and the algorithms are explained in section 2. In Section 3, the behavior of the models is shown and discussed. The paper is concluded in section 4.

## Methodology

### Dataset

**Fig. 1:**
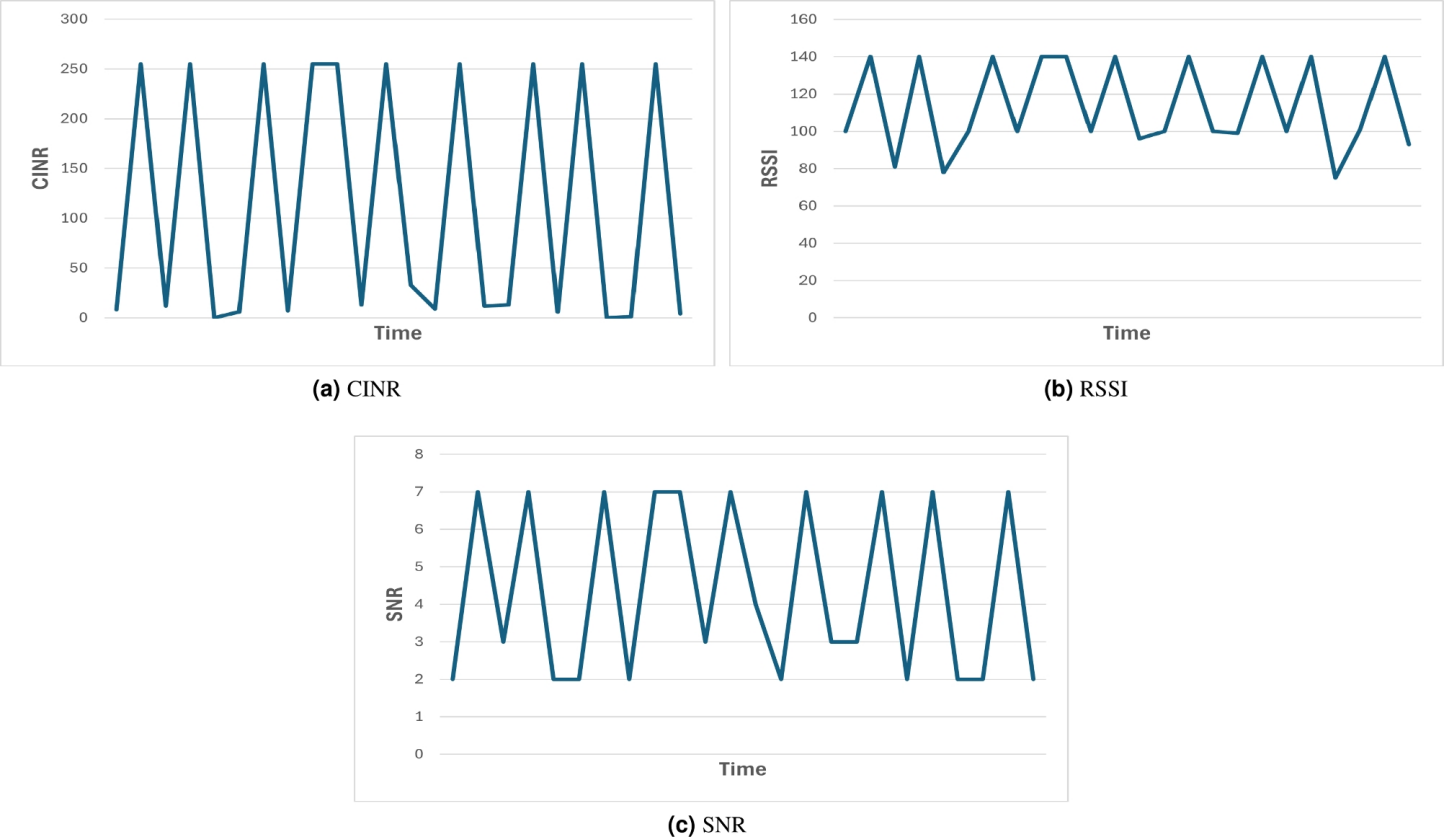
The values of the features of the dataset over time.

**Fig. 2:**
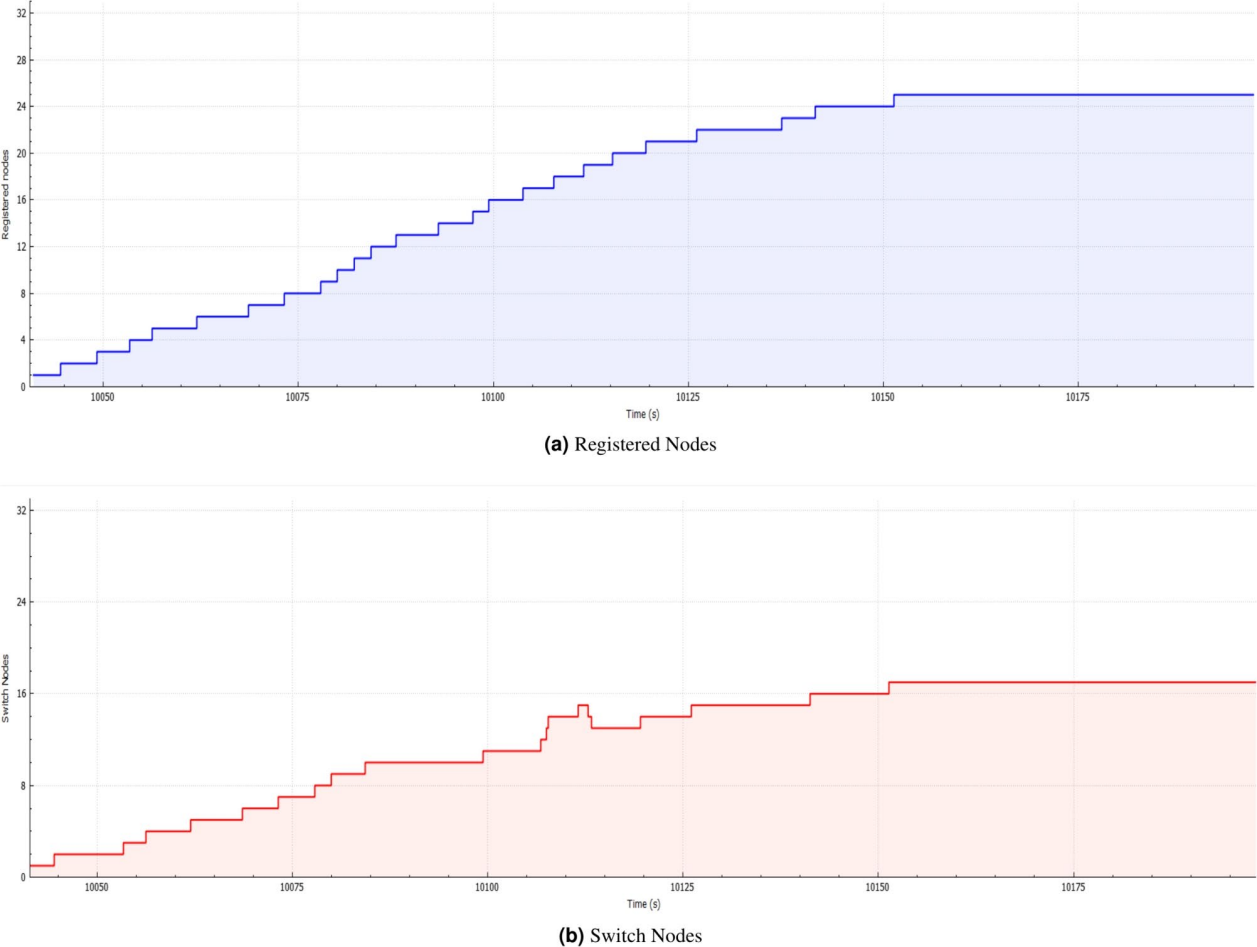
The activity of registered and switch nodes over Time.

The dataset consists of 2000 readings of a node in a network consisting of 500 nodes that was implemented by Microchip technology PL360 PLC transceiver using the PRIME standard. The dataset instances consist of CINR, SNR, and RSSI as the features and labels, which indicate whether the node has optimum reading or not, such that 1 represents ’UP’ and 0 represents ’Down’ as shown in Table 1. 50% of the data are for label 0 and the other 50% for label 1. The data is divided into 85% - 15% for the training and testing data, respectively. 15% of the training data has been passed to the semi-supervised learning models as unlabeled data. A subset of the dataset has been selected to plot the features over time, as shown in Figure 1. The correlation between the features has been analyzed, and as table 2 shows, there is a high correlation between the features. Figure 2 shows the number of registered nodes in a specific time instance such that the registered nodes are shown in Figure a and switch nodes are in Figure b. As Figure 2 shows, the more the number of registered nodes changes, the more the number of switch nodes changes.

**Table 1:** Sample of the Dataset.

SNR	CINR	RSSI	Label
2	0	75	0
3	13	99	1
2	0	75	0
3	16	100	−1
3	12	99	1

**Table 2:** Corelation Matrix between the dataset features.

	SNR	CINR	RSSI
SNR	1	0.975097572	0.923504485
CINR	0.975097572	1	0.941400059
RSSI	0.923504485	0.941400059	1

### The model

**Fig. 3:**
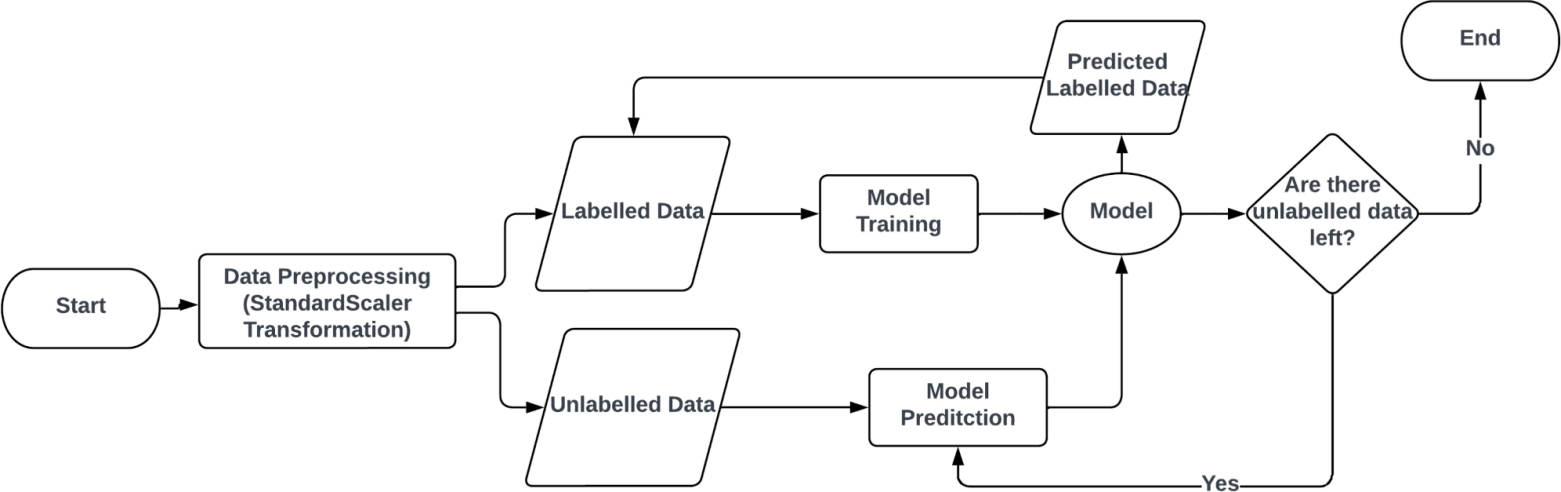
Model Flowchart.

**Fig. 4:**
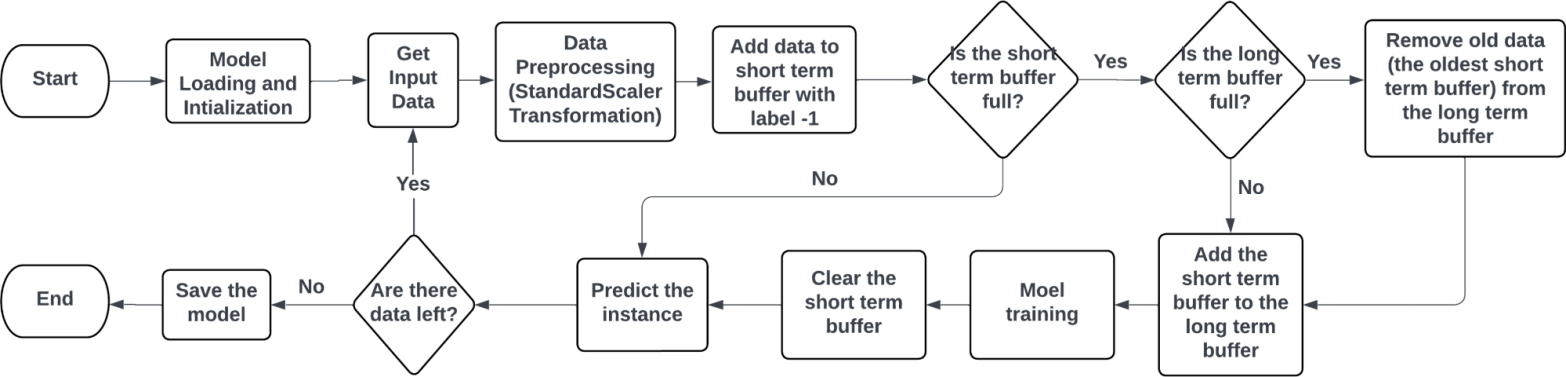
Flowchart of training of the model in real-life.

As Figure 3shows, the data are split into labeled data and unlabeled data. Then, the model is trained on the labeled data, then predicts a subset of the unlabeled data to be added to the labeled data to retrain the model, then it checks whether there are unlabeled data left or not, and the process iterates till all the unlabeled data are labeled. Semi-supervised Learning is divided into Transudative graph-based methods and inductive methods which is divided into wrapper methods, unsupervised preprocessing, intrinsically semi-supervised^29^. In this research, transudative graph-based methods and inductive wrapper methods are selected. Label Propagation, and Label Spreading representing the transudative graph-based methods and the wrapper methods are represented by Self-Training Classifier with Support Vector Machine (SVM) with linear and non-linear kernel, Light Gradieint Boosting Machine (LGBM) Label propagation algorithm and Label Spreading used K-Nearest Neighbours (KNN) such that labeled and unlabelled data as points on a graph such that the model labels the unlabeled data according to the nearby labeled points, but Label spreading adds regularization to avoid overfitting. Self-Training Classifier uses SVM and LGBM algorithms to predict the unlabeled data^30,31^. As a future work, the model shall be deployed on Raspberry Pi. The overview of the flow that the model is trained on is that batches of the data are collected in the short-term buffer, and all the batches are collected in the long-term buffer. Figure 4 shows the flow of the model when deployed in real life. The trained model is loaded, and long-term and short-term buffers are initialized. New data are processed and then added to the short-term buffer with −1 as a label. Then, checks if the short-term buffer is full; if true, it checks whether the long-term buffer is full or not. If it is full, it removes the oldest data, which is the oldest short buffer, and then the short buffer is added, and the model is trained. The short-term buffer is cleared, and then the instance is predicted. The model is saved if there is no data left and reloaded again after a constant period of time.

### Algorithms

#### Light Gradient-Boosting Machine (LGBM)

**Fig. 5:**
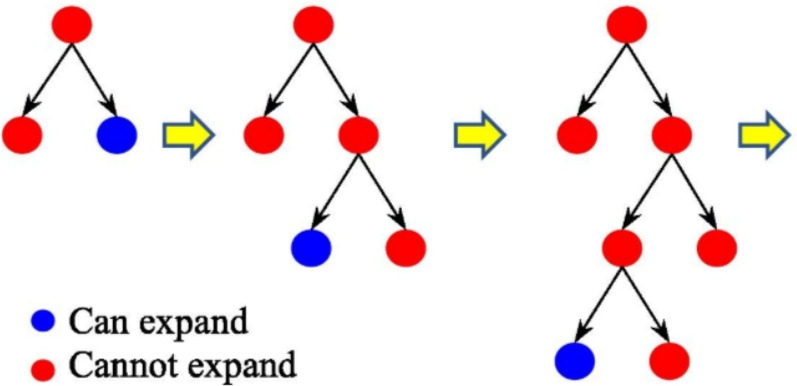
Leaf-wise Tree Growth^34^.

LGBM is a gradient boosting algorithm that depends on decision trees. The model is lightweight as it depends on Exclusive Feature Bundling (EFB) and Gradient-based One-Side Sampling (GOSS). EFB merges the mutually exclusive sparse features, so the number of features is reduced. GOSS selects the data instances that has large gradient to have high gain instead of scanning all data which lead to decreasing the data size, so the algorithm expands the decision tree leaf-wise as shown in Figure 5which leads to a faster training time up to twenty times compared to the conventional gradient boosting decision trees^32,33^.

#### Support Vector Machine (SVM)

**Fig. 6:**
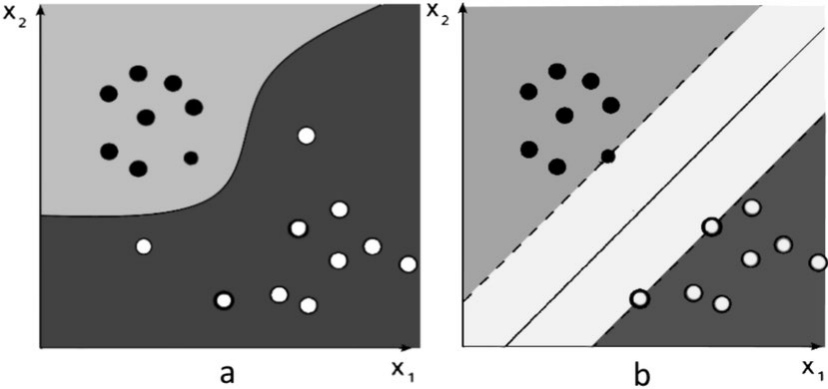
Non-linear and linear kernels of support vector machine^37^.

Support Vector Machine separates the data when they are not separable in their dimension into a high dimension. This separation divides them into classes, which is done using a kernel. The kernels can be a non-linear kernel, as shown in Figure 6 - a, and can be a linear kernel, as shown in Figure 6- b^35,36^.

#### K-Nearest Neighbors (KNN)

**Fig. 7:**
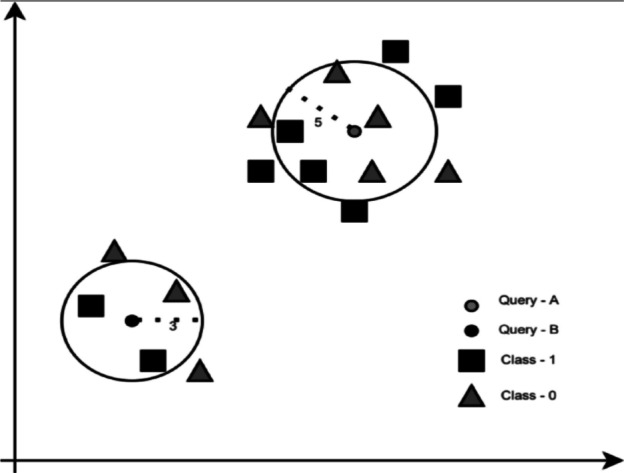
K-Nearest Neighbors^39^.

The KNN algorithm predicts the class of the input based on the most similar K points’ features. Figure 7shows the effect of choosing the value of k on the predicted class. When K=3, the input point is predicted as class 1 because between the nearest 3 points, the majority class is class 1. When K=5, the predicted class is class 0, as it is the majority class of the nearest 5 points. To have the best results, the model has to be tested for several values of Ks^38,39^.

#### Random forest

**Fig. 8:**
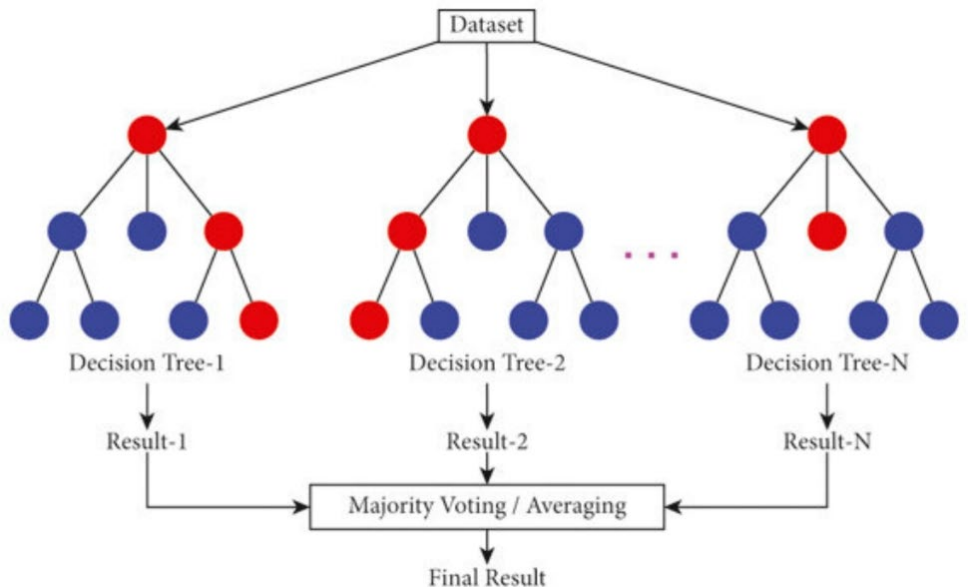
Random Forest Algorithm^42^.

Random Forest is an ensemble learning algorithm. As shown in Figure 8, it consists of decision trees such that each tree has a random subset of the data instances and features. Each tree gives a classification as a voting, and then the majority voting is the predicted class^40,41^.

#### Logistic regression

It is a method for binary classification. It predicts the probability of the occurrence of a class. It is the sigmoid function of the linear combination of features with weights as shown in equation (1)^43–45^.

1 $$\begin{aligned} \sigma \left( \sum _{i=1}^{n} w_i x_i + b \right) = \frac{1}{1 + e^{-\left( \sum _{i=1}^{n} w_i x_i + b \right) }} \end{aligned}$$

## Results

**Fig. 9:**
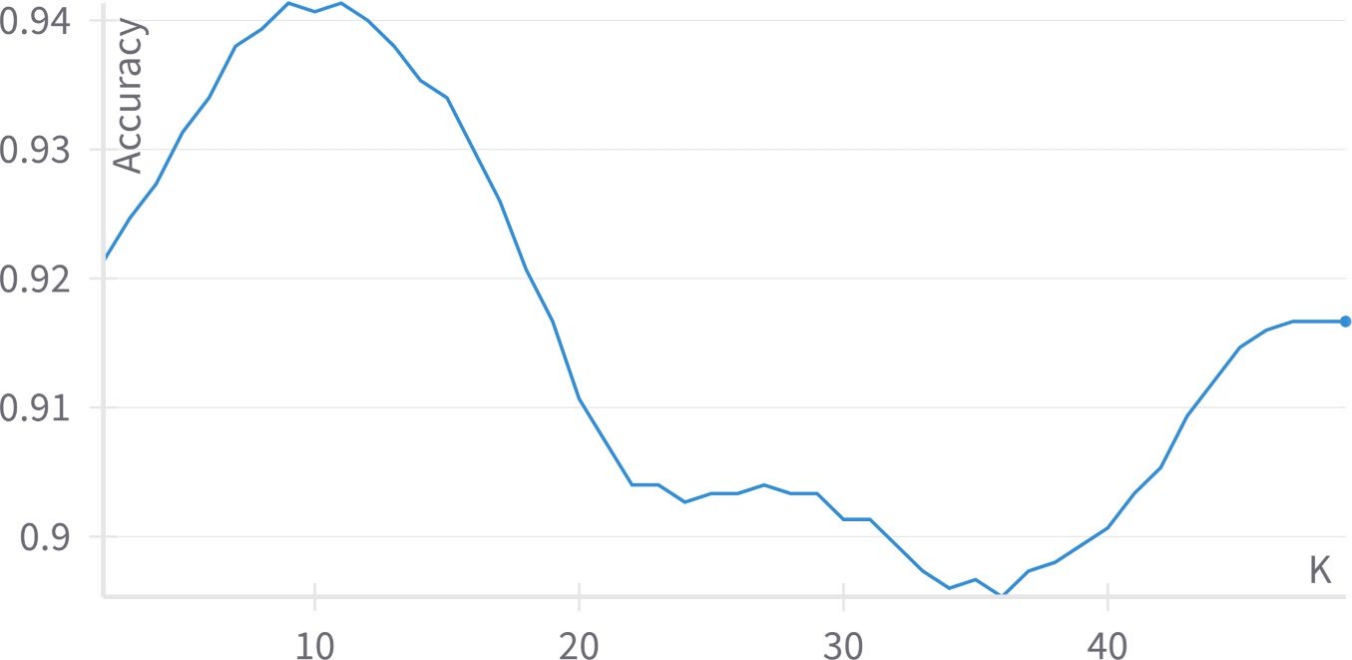
The effect of changing the value of K on the label propagation accuracy.

**Fig. 10:**
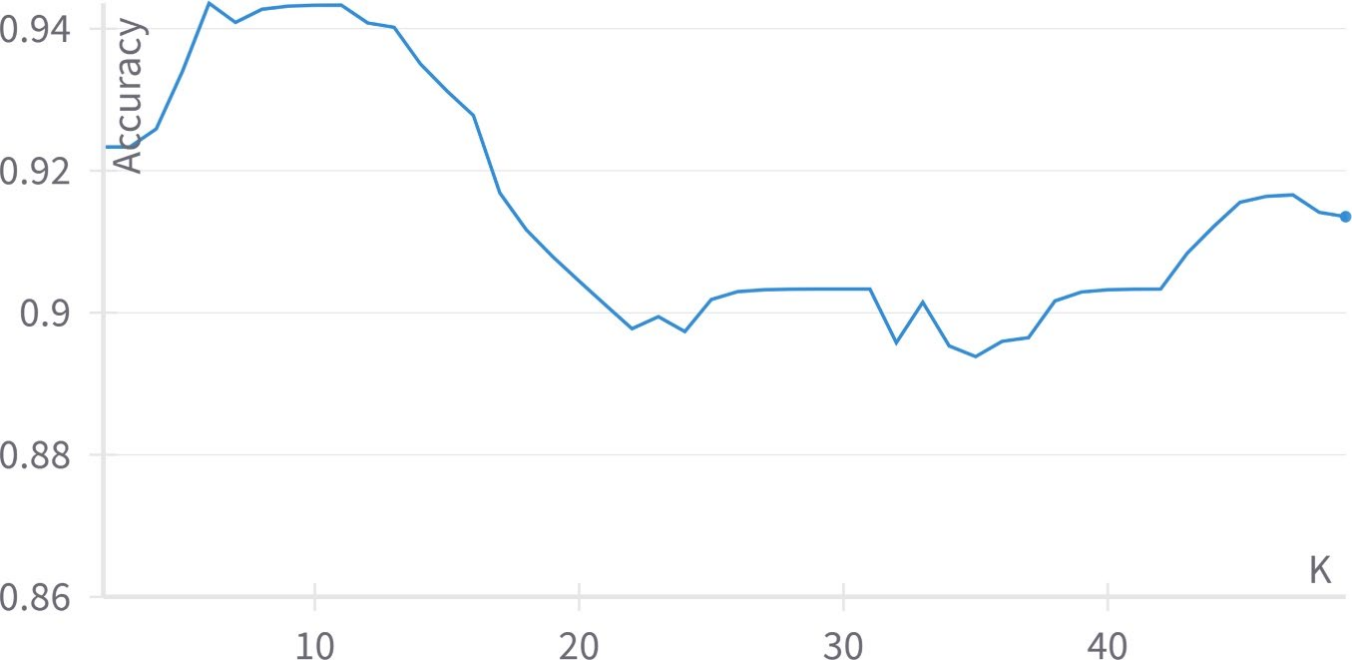
The effect of changing the value of K on the label spreading accuracy.

As table 3 shows, the best predictions are predicted by Label Spreading. Label Spreading has an average class accuracy equals 94.67%, F1-score equals 0.947, precision equals 0.946, the recall is 0.947, training time=0.018 sec. and the memory consumption is 0.99 MB. The least results resulted by a semi-supervised model were predicted by SVM with a linear kernel. It has an accuracy equals 90.33%, F1-score equals 0.903, precision equals 0.903, recall equals 0.904, training time=0.110 sec., and the memory consumption is 0.99 MB. The number of threads used for training the model has been limited to 4 as in the future, the model shall be deployed on a Raspberry Pi. The self-training classifier has 90% confidence, as the maximum iterations are adjusted to None such that it continues running till no unlabeled data are left. The label propagation and label spreading had the KNN kernel with K equals 9 and 6 respectively as shown in Figures 9 and 10 as a comparison between the different Ks has been conducted ranging between 2 and 49 to determine the best value of K resulting into the best accuracy.

### Light gradient-boosting machine

**Fig. 11:**
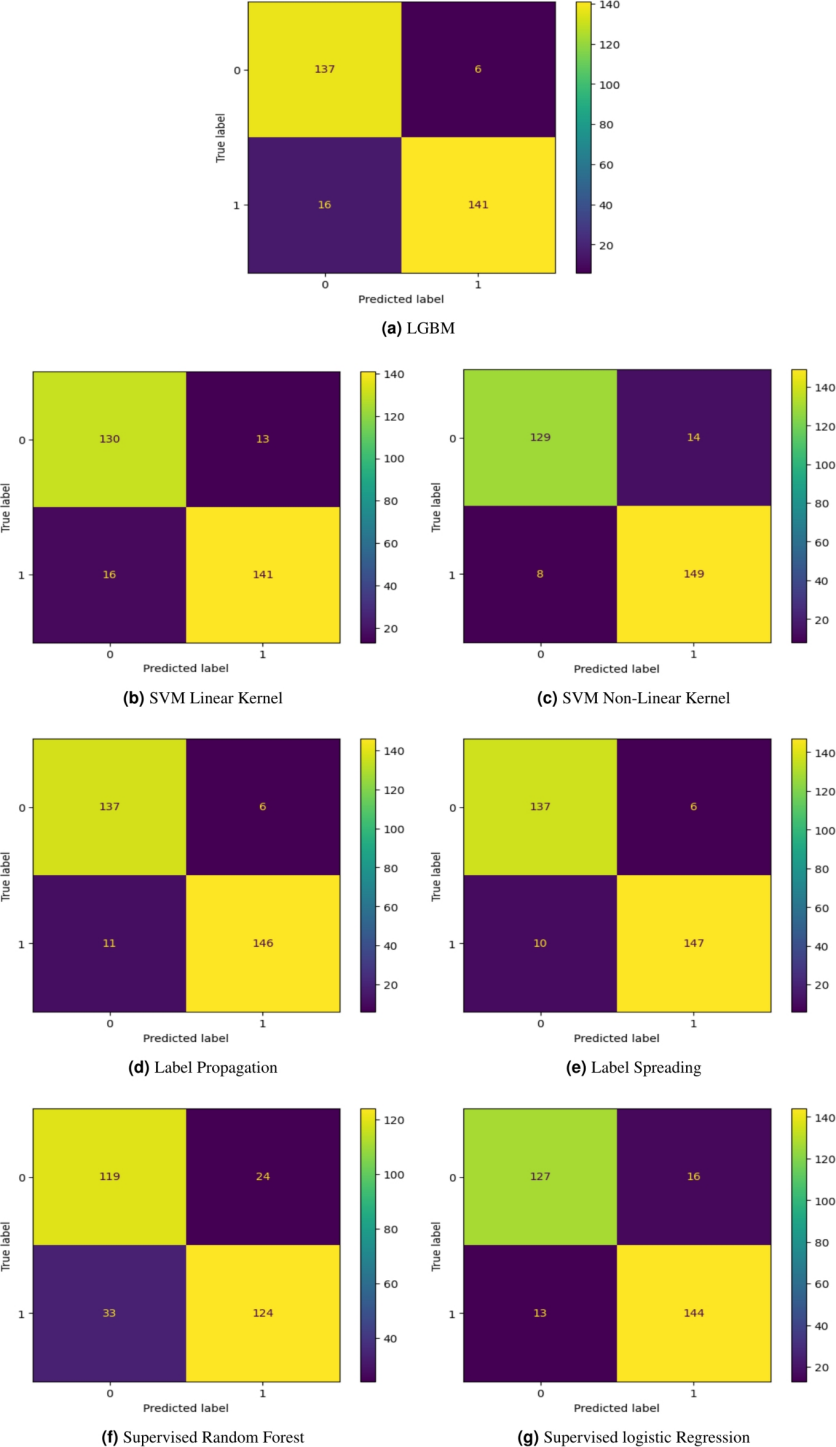
Confusion Matrices for the Trained Models.

Light Gradient-Boosting Machine predicted 137 instances out of 143 correctly for label 0 and 141 for label 157 for label 1 as Figure 11 - a shows.

### SVM

Support vector machine has been trained with linear kernel and non-linear kernel. Support vector machine using linear kernel predicted 130 instances out of 143 correctly for label 0 and 141 for label 157 for label 1 as Figure 11 - b shows. Figure 11 - c shows that the model predicted 129 out of 143 for label 0 correctly and 149 out of 157 correctly for label 1.

### Label propagation

The model predicted 137 instances for label 0 out of 143 correctly and 146 instances out of 157 for label 1, as Figure 11 - d shows.

### Label spreading

As Figure 11 - e shows, the model predicted 137 out of 143 instances correctly to predict label 0 and 147 out of 157 for label 1.

### Supervised random forest

It predicted 119 instances out of 143 correctly for label 0 and 124 for label 157 for label 1 as Figure 11 - f shows.

### Supervised logistic regression

It predicted 127 instances out of 143 correctly for label 0 and 144 for label 157 for label 1 as Figure 11 - g shows.

### Discussion

**Fig. 12:**
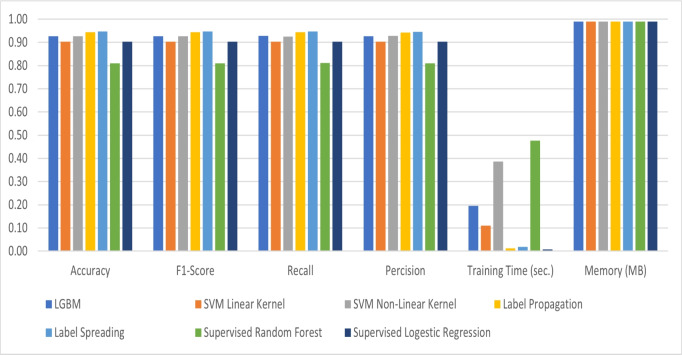
Comparison of the performance of the trained models.

As shown in Table 3 and Figure 12, the best accurate model, Label Spreading, is the most accurate model. It has a regularization phase, which deals with noise. It has an accuracy of 94.67%, an f1-score equals 0.947, a precision is 0.946, and recall equals 0.947 with training time equals 0.018 sec. and memory consumption equals 0.99 MB. A couple of Supervised Learning models have been trained on the same dataset (excluding the unlabeled data) to be compared with the semi-supervised learning models. The supervised learning models are Random Forest and Logistic Regression. Random Forest has an accuracy of 81%, an f1-score equals 0.81, a precision equals 0.81, a recall equals 0.811, a training time is 0.477 seconds, and memory consumption equals 0.99 MB. Logistic Regression’s accuracy is 90.33%, the f1-score is 0.903, precision is 0.904, recall is 0.903, training time equals 0.007 sec, and the memory consumption is 0.99 MB. Both models have yielded lower results than the trained semi-supervised learning models. Although the logistic regression has the least training time, it yielded 90.33% accuracy, while the best semi-supervised learning trained model, which is the label spreading, resulted in 94.67%.

**Table 3:** Numeric results of the trained models.

Algorithm	Accuracy	F1-Score	Recall	Percision	Training Time (sec.)	Memory (MB)
LGBM	92.67%	0.927	0.928	0.927	0.195	0.99
SVM Linear Kernel	90.33%	0.903	0.904	0.903	0.110	0.99
SVM Non-Linear Kernel	92.67%	0.926	0.926	0.928	0.386	0.99
Label Propagation	94.33%	0.943	0.944	0.943	0.012	0.99
Label Spreading	94.67%	0.947	0.947	0.946	0.018	0.99
Supervised Random Forest	81.00%	0.810	0.811	0.810	0.477	0.99
Supervised Logestic Regression	90.33%	0.903	0.903	0.904	0.007	0.99

## Conclusion

PLC communication is the transfer of data using power lines. PLC nodes face conditions that make it unavailable in some time slots. Detecting these time slots earlier saves time. Machine learning can do this detection, but the problem is that the availability of PLC nodes changes due to the surrounding environment, including noise, collisions, or power outages. False predictions in real life, when data is sent to an unavailable node, time and power are consumed as the data are retransmitted to another node. An AI model needs to be trained continuously to better predict the availability of a node. In this paper, a semi-supervised machine learning approach has been introduced. Label propagation, label spreading, and self-training classifier have been used with Light Gradient Boosting Machine and Support Vector Machine (linear and non-linear kernel) algorithms. The algorithms were trained on a dataset consisting of 2000 instances of CINR, SNR, and RSSI as features and node up/down as a label such that 85% of the data are training set and the rest are testing set. 15 % of the training set were unlabeled. Label Spreading had the best results with an accuracy of 94.67%, f1-score equals 0.947, precision is 0.946, and recall equals 0.947 with training time equals 0.018 sec. and memory consumption equals 0.99 MB. The models have been compared to the supervised learning Random Forest and Logistic regression. Logistic Regression performed better than the Random Forest but had less accurate results than the label spreading, achieving an accuracy of 90.33%, an f1-score equals 0.903, precision is 0.904, recall is 0.903 with training time equals 0.007 sec. and memory consumption equals 0.99 MB. As a future work, the model shall be deployed on a Raspberry Pi.

## Data Availability

The datasets generated during and/or analyzed during the current study are available from the corresponding author upon reasonable request.
